# MENTORSHIP AND CAREER PATHS IN GEROPSYCHOLOGY

**DOI:** 10.1093/geroni/igz038.039

**Published:** 2019-11-08

**Authors:** Lindsey Jacobs, Rebecca S Allen, Jessica Strong, Elizabeth Shumaker

**Affiliations:** 1 VA Boston Healthcare System, Brockton, Massachusetts, United States; 2 University of Alabama, Tuscaloosa, Alabama, United States; 3 San Francisco VA Health Care System, San Francisco, California, United States

## Abstract

Mentoring is a critical process for guiding the next generation of geropsychologists into career paths that fit their goals and needs. However, the context and content of mentorship in geropsychology is not well-documented. Understanding geropsychologists’ experiences may provide some insight into how mentorship is executed in academia versus clinical settings and which factors are relevant to career decision-making. We conducted a qualitative analysis of 28 transcripts from individual telephone interviews with geropsychologists and geropsychology trainees, focusing on mentorship and how it influenced their career path. Themes were differences in the focus of mentorship (e.g., immediate projects versus career goals), frequency of career-focused mentorship, length of mentorship (years versus months), and mentors’ openness to share opinions and experiences (e.g., academic mentors are more open about negative aspects of academia, while clinical mentors “guard” trainees from the negative aspects of clinical work). Subthemes and recommendations will be discussed.

